# A Case of Transfusion of Blood

**Published:** 1889-05

**Authors:** P. J. Keegan

**Affiliations:** Albany, N. Y.


### A CASE OF TRANSFUSION OF BLOOD.

BY P. J. KEEGAN, M. D., ALBANY, N. Y.

March 5, 1878, A. B., a well developed young man of excellent family and personal history and habits, ate lunch (a small pie) at 10 A. M. Before 12 M. was taken with severe pain, as in biliary colic; was obliged to go to stool, and had a copious fluid movement. Had three such evacuations within half an hour; became weak, was carried home, and had another (the fourth) evacuation, very similar to the others, according to his judgment. This time he used a chamber, and found that the evacuation was blood, and concluded that the others were also blood, a fact which he had failed to note, as he had used a water-closet at his office, and each time emptied the basin before rising.

I saw the man before one o'clock P. M., and found him very exsanguinated and weak; heart weak, bounding and rapid; temperature low (now recorded); tongue and all visible mucous membrane nearly clear white from want of blood. The diagnosis was "passage of gall-stone, with rupture of the common duct," thus endeavoring to account for the pain and hemorrhage. The usual drugs were given to relieve the pain and bleeding, which gradually ceased.

Five days after the first attack, a feeling of distress was noticed in the region of the gall-bladder, the stools became clayey, the skin became gradually more and more jaundiced, the urine darker, and a small tumor was discovered beneath the liver and attached to that organ. "Occlusion of the common gall-duct, with distention of the gall-bladder," was now the condition supposed. In this I was supported by Dr. Ward and others who saw the case. The gall-bladder continued to increase, and all the other symptoms became more marked until about the 16th. At that time I was about to tap the gall-bladder, its lower extremity reaching the patient's umbilicus, when I noticed that it was smaller, less tense and less sensitive than on the previous day. The color of the stools became dark, and all things pointed to a re-establishment of the flow of bile by the natural passage. He now continued to improve until the 24th, when he was able to walk and did walk a little in his house. He took more exercise than he was able to bear, became exhausted, and was necessarily assisted to his bed again. About 9 P. M. of the 25th his bleeding again returned, and what was closely estimated at four pints was voided. This blood came in such form that his friends supposed it to be mixed with a great portion of his intestines, and so reported it to me. They emptied the vessel, so I did not know of what the discharge consisted until about 7 P. M. of the 26th, when I was called, and found a large chamber nearly full (about two quarts) of blood mixed with what I at first was sure was portions of bowel. Examination proved this to be casts of the bowels, of wonderful accuracy, composed of blood fibrin, the pieces or casts broken into lengths of from three inches to three feet. He was again exceedingly exsanguinated, and so prostrated as to be utterly helpless, even appearing unable to move or speak.

On the 27th, he was apparently dying; no pulse at wrists, cold to knees and elbows; cold perspiration on extremities and face; heart weak and fluttering, 150 beats to minute; sensation so very obtuse that he did not notice pain from the very careful dissection which I was obliged to make to find a vein in his arm. The arm was ligated above the elbow, in hopes to fill a vein, that it might be seen for a guide, but none could be detected, and a slow cutting search was resorted to. A cut one inch long was made diagonally across to line of the median basilic, and when the vein was found, it could only be verified by puncturing it and permitting a drop of venous blood to escape into the wound. During the dissection for the vein, only *about two drops of blood* oozed into the wound.

About twenty ounces of blood were taken from the arm of a healthy young man, defibrinated by whipping with broom corn, and strained through a silk handkerchief. Care was taken that the utensils used during the manipulations were scrupulously clean. The temperature of the blood was kept at 102° F. Twelve ounces of the defibrinated blood were taken into a Dieulafoy aspirator, the tubes carefully heated to the required temperature, the needle thrust into the exposed vein, and the blood slowly injected into the circulation, eight ounces being given. The stimulating effect of the blood transfused was most gratifying. The work was not completed when the patient spoke, saying, "I feel better." He had not spoken to me that morning, and, while conscious, was unequal to the exertion required in speaking. From the time of the transfusion the hemorrhages ceased, and he gradually but slowly made a good recovery.

To illustrate his extreme lack of vitality, the edges of the wound in the arm did not become reddened, nor was there any evidence of repair or healing of the parts until the third week.—*Albany Med. Annals.*

